# Supplementary figures and images for: The Beta-Tubulin Isotype TUBB6 Controls Microtubule and Actin Dynamics in Osteoclasts
Source: Front Cell Dev Biol. 2021 Nov 18;9:778887. doi: 10.3389/fcell.2021.778887 (PMC8639228; doi:10.3389/fcell.2021.778887)

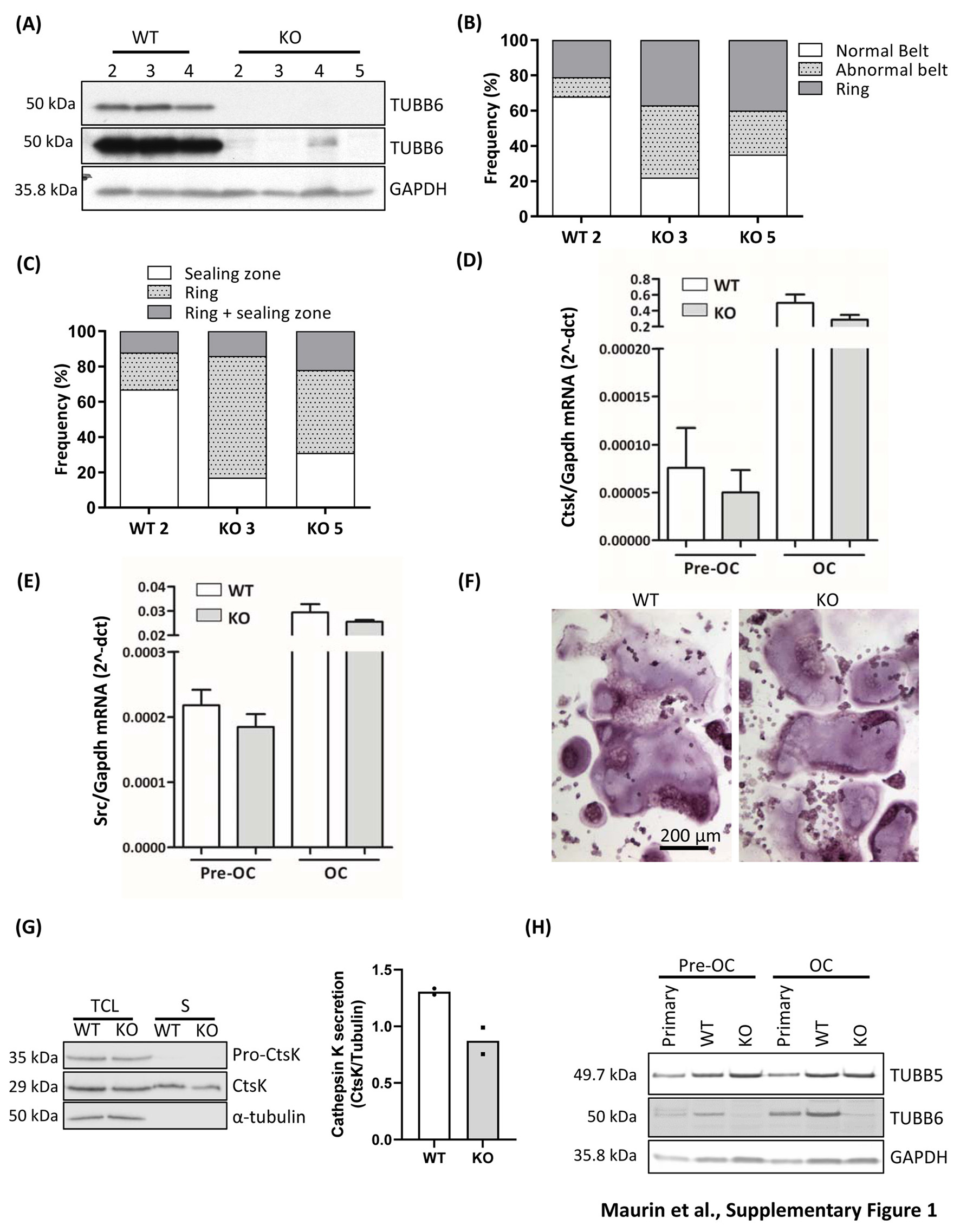

Supplement: Supplementary Figure 1 — (A) Representative immunoblot showing TUBB6 and GAPDH expression in WT and Tubb6 KO RAW264.7 clones. Note in overexposed panel in the middle that clones 2 and 4 retain traces of TUBB6. (B) Bar graph showing the frequency of WT and KO osteoclasts seeded on glass and presenting podosome rings, normal or abnormal podosome belt, counting a total of 200 WT and KO osteoclasts in two independent experiments. (C) Bar graph showing the frequency of WT and KO osteoclasts seeded on ACC and presenting sealing zone and/or rings counting over 50 osteoclasts per clone. (D,E) Bar graphs showing the level of osteoclast characteristic Cathepsin K (D) and Src (E) mRNAs relative to Gapdh as determined by Q-PCR in WT or Tubb6 KO RAW264.7 cells (Pre-OC) or osteoclasts (OC). (F) Representative images showing TRAP staining in WT and Tubb6 KO osteoclasts. (G) Representative immunoblot blot showing intracellular content of alpha-tubulin, Cathepsin K proenzyme, and its mature form in total cell lysates of WT and Tubb6 KO osteoclasts, and the secretion of mature Cathepsin K in the culture medium of the same cells. (H) Representative immunoblot blot showing TUBB5, TUBB6 and GAPDH expression in primary bone marrow macrophages (Pre-OC) and osteoclasts (OC) and in WT or Tubb6 KO RAW264.7 cells (pre-OC) or osteoclasts (OC). [file Image_1.JPEG]

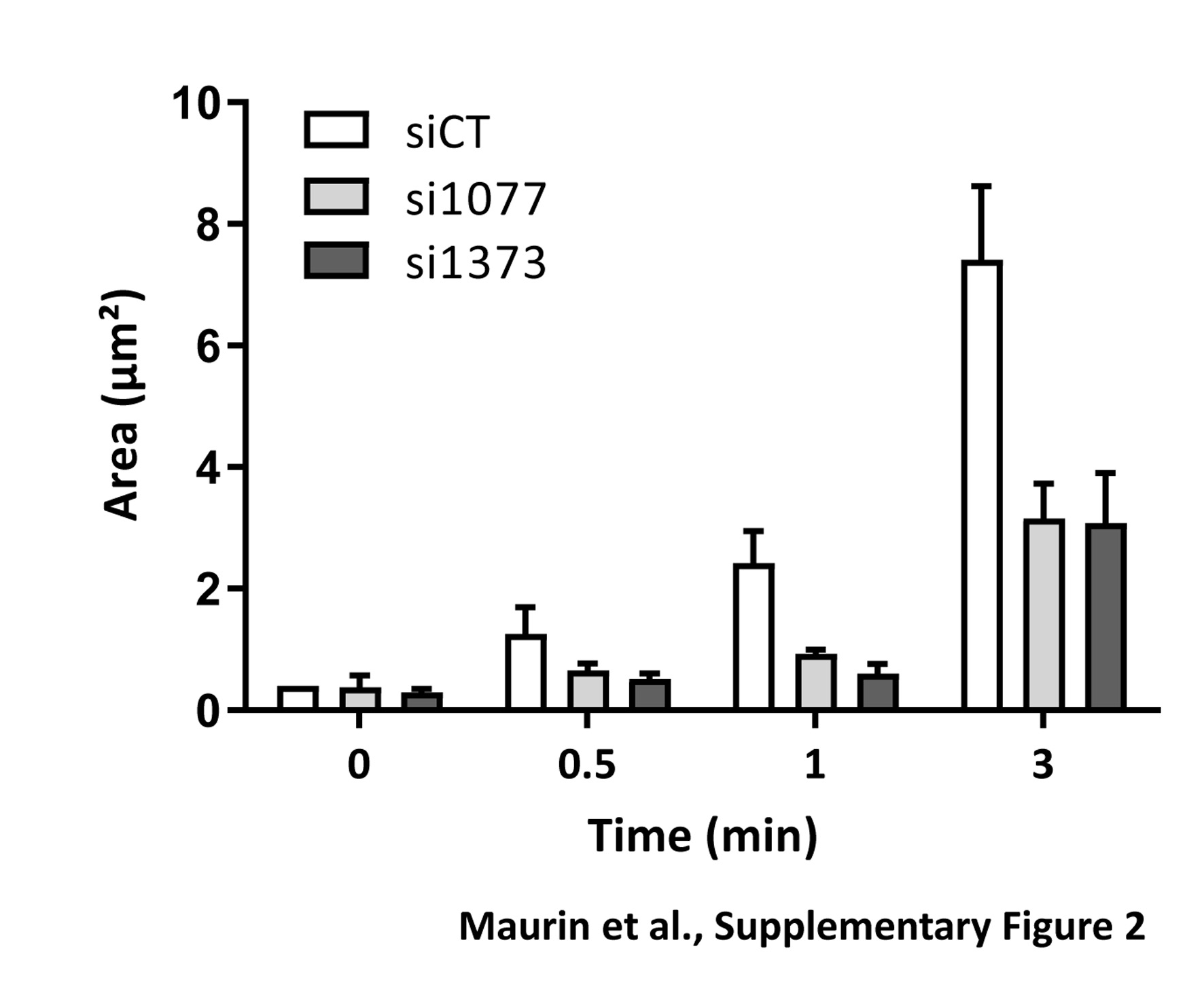

Supplement: Supplementary Figure 2 — Bar graph showing mean and SEM α-tubulin aster area after nocodazole washout in osteoclasts transfected with luciferase control siRNA or Tubb6 siRNAs si1077 and si1373, measuring at least 20 asters per group were measured in four independent experiments. [file Image_2.JPEG]

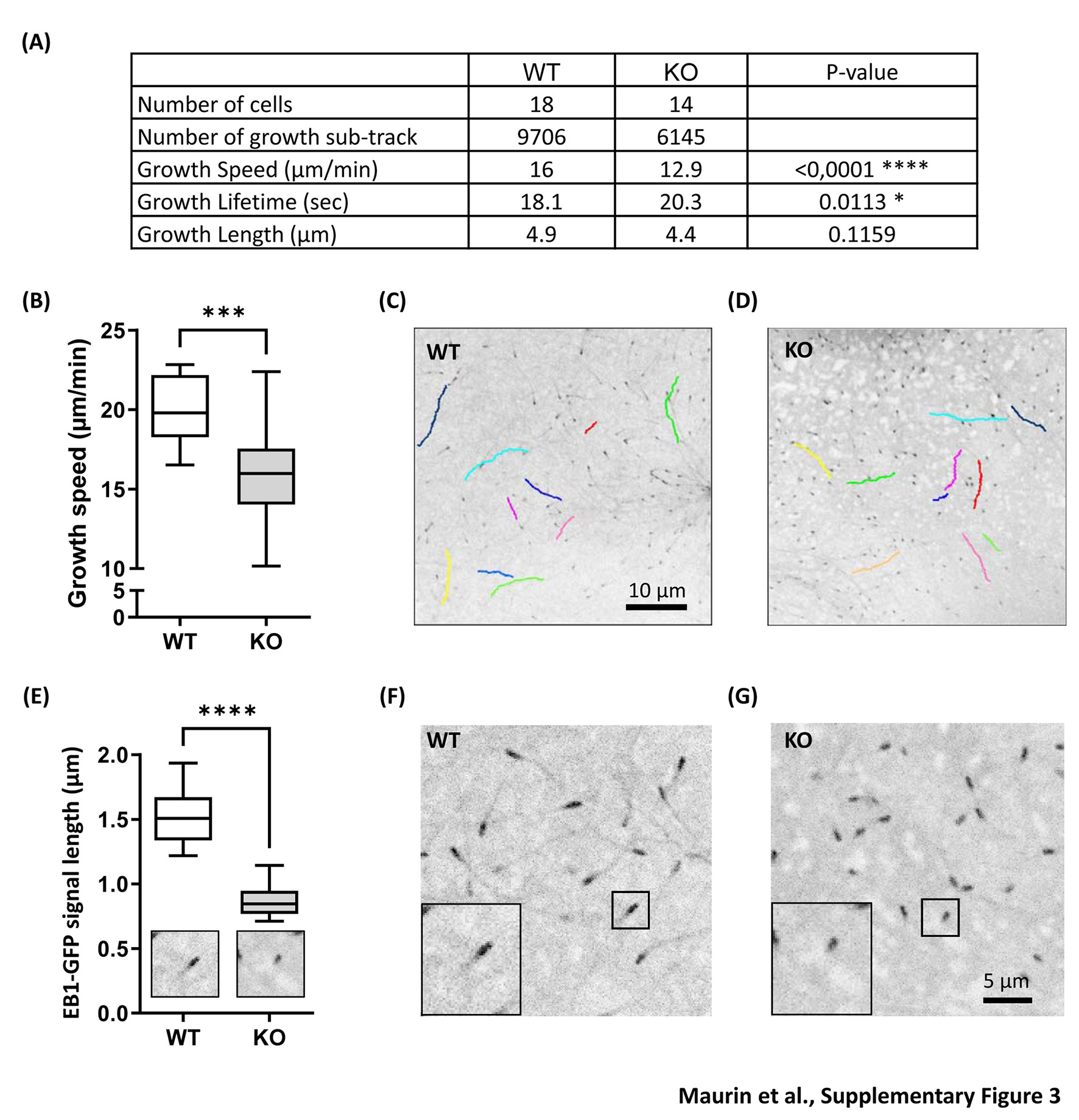

Supplement: Supplementary Figure 3 — (A) Table showing the details of microtubule dynamics parameters from u-track analysis (B) Minimum to maximum boxplot showing microtubule growth speed determined in ImageJ 1.53c with MTrackJ in 15 WT and Tubb6 KO osteoclasts expressing EB1-GFP from three independent experiments and measuring 10–11 comets per osteoclasts; Mann–Whitney test: ***p < 0.0002. (C,D) Representative EB1-GFP comets tracks in WT (C) and Tubb6 KO (D) analyzed in (B). (E) Minimum to maximum boxplot showing the length of the EB1-GFP signal in 18 WT and Tubb6 KO osteoclasts per condition from three independent experiments and measuring 25 comets in each osteoclast to determine the average comet length per osteoclast; Mann–Whitney test: ****p < 0.0001. (F,G) Representative images of the EB1-GFP signal at the tip of microtubules in WT (F) and KO (G) osteoclasts analyzed in (E). [file Image_3.JPEG]

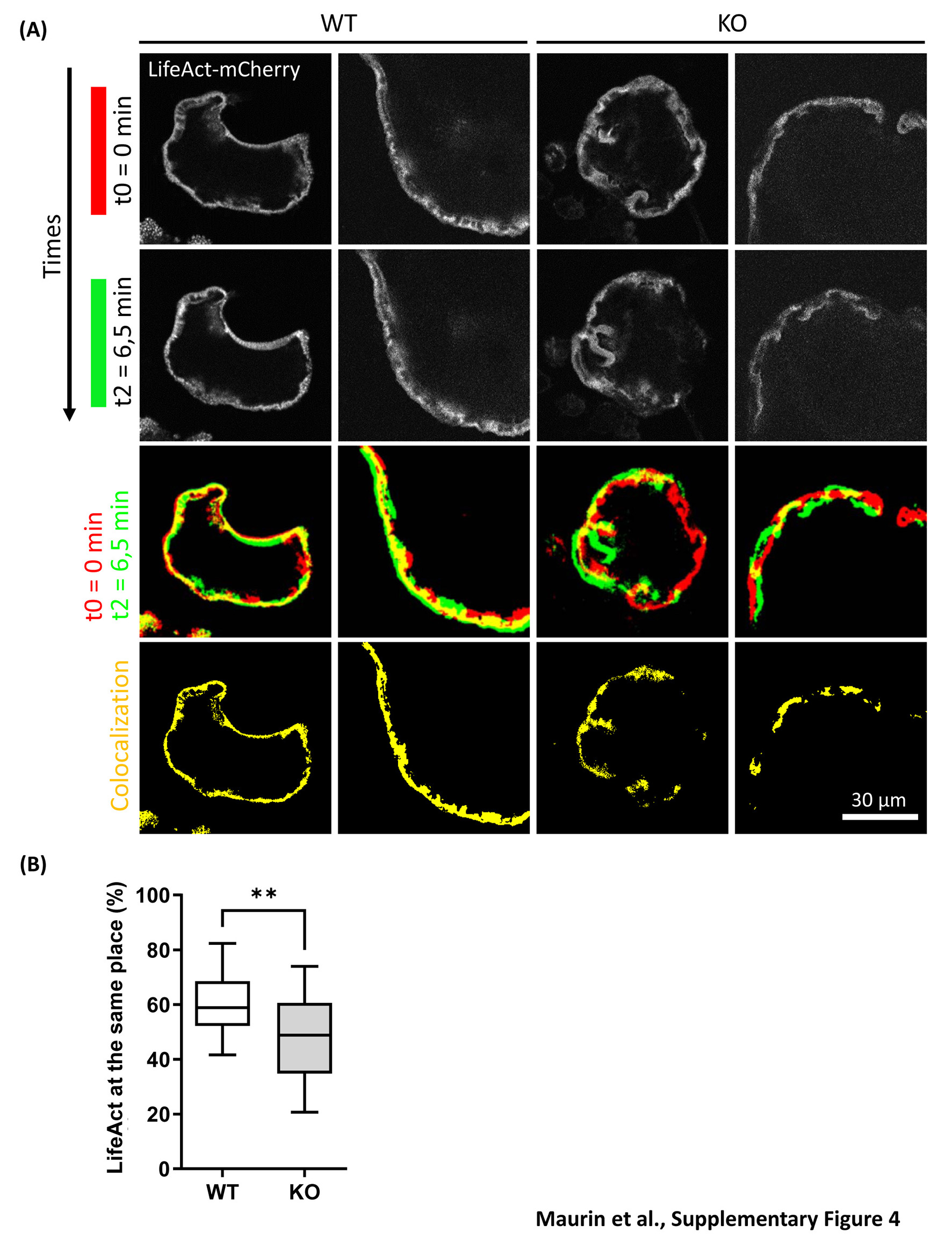

Supplement: Supplementary Figure 4 — (A) Representative live confocal images of osteoclasts derived from WT or Tubb6 KO osteoclasts expressing LifeAct-mCherry and sitting on glass, showing the localization of LifeAct-mCherry signal at t0 = 0 min (red) and t2 = 6.5 min (green), with the overlapping areas in yellow. (B) Minimum to maximum boxplot showing the percentage of LifeAct-mCherry signal at t0 that persists at the same position at t2 in WT or Tubb6 KO osteoclasts; in a total of 22 WT and 21 KO osteoclasts from four different experiments. Mann–Whitney test: **p < 0.01. [file Image_4.JPEG]
